# Hinokiflavone Inhibits MDM2 Activity by Targeting the MDM2-MDMX RING Domain

**DOI:** 10.3390/biom12050643

**Published:** 2022-04-27

**Authors:** Viktoria K. Ilic, Olga Egorova, Ernest Tsang, Milena Gatto, Yi Wen, Yong Zhao, Yi Sheng

**Affiliations:** 1 Department of Biology, York University, Room 327B Life Science Building, 4700 Keele Street, Toronto, ON M3J 1P3, Canada; viktoria.ilic@gmail.com (V.K.I.); olga-egorova@hotmail.com (O.E.); ernesttsang@hotmail.com (E.T.); milenagatto555@gmail.com (M.G.); yiwen15@my.yorku.ca (Y.W.); 2 Beijing Computing Center, Beijing Academy of Science and Technology, Beijing, 100094, China; yongzhao168@gmail.com

**Keywords:** MDM2, E3 ligase inhibitor, p53, MDMX, ubiquitination

## Abstract

**Simple Summary:**

MDM2 is a ubiquitin E3 ligase, frequently overexpressed in human cancers. A biflavonoid Hinokiflavone was identified by virtual screening to bind the MDM2-MDMX RING domain and inhibit MDM2 E3 ligase activity. Hinokiflavone was shown to downregulate MDM2 and its homolog protein MDMX and inhibit the tumorigenic activity of MDM2 in cancer cells. Hinokiflavone could work as a potential anti-cancer therapeutic agent in malignancies with MDM2 overexpression.

**Abstract:**

The proto-oncogene *MDM2* is frequently amplified in many human cancers and its overexpression is clinically associated with a poor prognosis. The oncogenic activity of MDM2 is demonstrated by its negative regulation of tumor suppressor p53 and the substrate proteins involved in DNA repair, cell cycle control, and apoptosis pathways. Thus, inhibition of MDM2 activity has been pursued as an attractive direction for the development of anti-cancer therapeutics. Virtual screening was performed using the crystal structure of the MDM2-MDMX RING domain dimer against a natural product library and identified a biflavonoid Hinokiflavone as a promising candidate compound targeting MDM2. Hinokiflavone was shown to bind the MDM2-MDMX RING domain and inhibit MDM2-mediated ubiquitination in vitro. Hinokiflavone treatment resulted in the downregulation of MDM2 and MDMX and induction of apoptosis in various cancer cell lines. Hinokiflavone demonstrated p53-dependent and -independent tumor-suppressive activity. This report provides biochemical and cellular evidence demonstrating the anti-cancer effects of Hinokiflavone through targeting the MDM2-MDMX RING domain.

## 1. Introduction

The tumor suppressor protein p53 is inactivated by mutations of the *TP53* gene in over 50% of human primary cancers [1,2]. However, in many cancer types, such as leukemia, sarcoma, and melanoma, genetic mutations of *TP53* are less common, and p53 inactivation is often caused by alterations in p53-regulatory proteins, in particular by amplification and overexpression of MDM2 (HDM2 in humans) and MDMX (HDM4 or HDMX in humans) [1,3,4,5,6,7].

MDM2 and MDMX are the key negative regulators of p53. MDM2 functions as the E3 ubiquitin (Ub) ligase targeting p53 for ubiquitination-mediated proteasomal degradation [8,9,10]. MDMX is an MDM2 homolog. Despite lacking E3 ligase activity on its own, MDMX heterodimerizes with MDM2 to enhance MDM2-mediated p53 ubiquitination [11,12,13]. Both MDM2 and MDMX can interact with p53 to inhibit its transcriptional activity [13]. Thus, aberrant regulation of MDM2 and MDMX in cancer cells is a key mechanism of p53 inactivation and represents an important therapeutic target in many types of cancers [11,14].

In addition to suppressing the function of p53, increasing evidence suggests that MDM2 possesses p53-independent oncogenic potential [15]. MDM2 was shown to inhibit the tumor suppressors Rb and Foxo3a [16,17,18], altering cell cycle progression [19,20], and interfering with DNA replication and DNA repair [21,22,23]. The tumorigenic potential of MDM2 was further demonstrated by its ability to transform rodent fibroblasts, and to promote tumor formation and progression in nude mice or Eu-myc transgenic mice [24,25,26,27]. This inspired us to search for novel MDM2 inhibitors that could abrogate MDM2 E3 ligase activity and downregulate the activity of MDM2-MDMX in cancer cells.

MDM2 E3 ligase activity relies on homo-dimerization through its own RING domain or hetero-dimerization with MDMX. The MDM2-MDMX heterodimer was shown to be a more stable complex and the primary form for the negative regulation of p53 in cells [28,29]. Using the crystal structure of the MDM2-MDMX RING domain dimer as a template [12], we performed an in silico screening against a natural product library and identified the biflavonoid Hinokiflavone as a candidate MDM2 inhibitor. Remarkably, Hinokiflavone inhibited MDM2 E3 ligase activity and caused the downregulation of MDM2 and MDMX in the tested cancer cell lines. Hinokiflavone demonstrated p53-dependent and -independent tumor-suppressive activity, strongly suggesting its therapeutic potential as an anti-cancer compound through inhibition of MDM2 and MDMX.

## 2. Results

### 2.1. Hinokiflavone Interacts with the MDM2-MDMX RING Domain In Vitro

**Figure 1:**
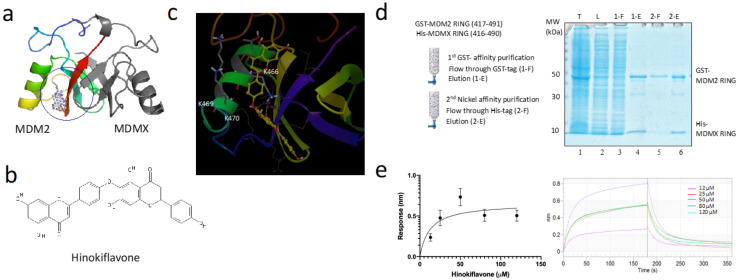
Hinokiflavone interacts with the MDM2-MDMX RING domain dimer. (**a**) The cartoon representation of the MDM2-MDMX RING domain dimer, with the MDM2 RING domain and the MDMX RING domain shown in rainbow and grey, respectively. The surface pocket of the MDM2-MDMX RING heterodimer used for in silico screening was shown in the space-filled model. (**b**) Chemical structure of Hinokiflavone. (**c**) The docking model of Hinokiflavone bound with the MDM2-MDMX RING dimer. Hinokiflavone is shown in ball and stick and forms contacts with MDM2 residues K466, K469, and K470. (**d**) MDM2-MDMX RING dimer was purified using a sequential two-step GST and nickel affinity method. (**e**) The physical interaction between Hinokiflavone and the MDM2-MDMX RING dimer was examined by BLI. (**e**, **left**) The BLI signals (nm) of the immobilized MDM2-MDMX RING dimer with the addition of Hinokiflavone (0–120 μM). (**e**, **right**) The BLI ligand association and dissociation plots of the MDM2-MDMX RING dimer with the addition of Hinoflavone (0–120 μM).

MDM2 is a RING domain E3 ligase, which uses its RING domain to recruit a Ub conjugating enzyme E2 and transfer the Ub from the E2 to a lysine residue on MDM2 (autoubiquitination) or the target substrate protein (substrate ubiquitination) [12]. We performed structure-based virtual screening using the crystal structure of the MDM2-MDMX RING domain dimer (PDB:2VJE) to identify compounds that might potentially bind MDM2 and inhibit its E3 ligase activity (Figure S1). Hinokiflavone (4′, 6″-O-Biapigenin) was identified as one of the top-ranking chemicals, which targets a cleft close to the MDM2-MDMX dimerization interface (Figure 1a–c, Figure S2). The docking model revealed that Hinokiflavone inserts into a groove between the α-helix and β3 of the MDM2 RING domain, making contacts with the residues from MDM2 α1 and β3 and the residues from MDMX β2 (Figure S2).

To test the interaction between the MDM2-MDMX RING domain dimer and Hinokiflavone, we purified the heterodimer of the GST-tagged MDM2 RING domain and the His-tagged MDMX RING domain through a sequential GST- and nickel affinity purification (Figure 1d). We then examined the binding of Hinokiflavone to the MDM2-MDMX RING domain dimer using a Biolayer Interferometry (BLI) assay. Hinokiflavone was shown to interact with the MDM2-MDMX RING domain dimer with an affinity of 12 μM (Figure 1e).

### 2.2. Hinokiflavone Inhibits the MDM2 RING Domain-Mediated Ubiquitination

**Figure 2:**
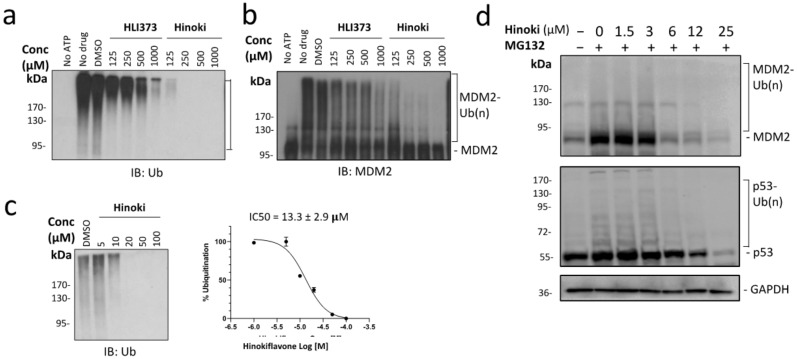
Hinokiflavone inhibits MDM2-mediated ubiquitination. (**a**,**b**) Ubiquitination activity of MDM2 was studied in the presence of Hinokiflavone or MDM2 inhibitor HLI373 using in vitro ubiquitination assay. In this assay, polyubiquitination was detected by an anti-Ub antibody (**a**). MDM2 ubiquitination was detected using an anti-MDM2 antibody (**b**). (**c**) MDM2 in vitro ubiquitination assay to determine Hinokiflavone IC_50_. (**c**, **left**) The MDM2 in vitro ubiquitination assay was performed in the presence of Hinokiflavone (0–100 μM). (**c**, **right**) The ubiquitination levels were quantified by densitometry using Fiji. The inhibitory effect of Hinokiflavone was plotted and IC_50_ was calculated using Prism 9.0. (**d**) Hinokiflavone attenuated MDM2 and p53 ubiquitination in AML-2 cells. AML-2 cells were treated with Hinokiflavone (0–25 μM) for 24 hrs followed by 4 hrs treatment with MG132 (10 μM). MDM2 and p53 were detected by immunoblotting with MDM2 and p53-specific antibodies. GAPDH was used as a loading control.

To examine whether Hinokiflavone inhibits MDM2 E3 ligase activity, an in vitro ubiquitination assay was performed, in which ATP, E1, E2, Ub, and MDM2 were provided to facilitate MDM2 ubiquitination, and candidate compounds or DMSO (vehicle control) were added to examine their ability to block MDM2 E3 ligase activity. Hinokiflavone was found to inhibit MDM2 ubiquitination with an IC_50_ value of ~13 μM, which is more potent than a known MDM2 RING domain antagonist HLI373 (Figure 2a–c) [30].

To determine the effect of Hinokiflavone on MDM2 and p53 ubiquitination in the cell, the proteasome inhibitor MG132 was employed to enrich the cellular ubiquitinated MDM2 and p53 using a leukemia cell line AML-2. When AML2 cells were treated with increasing doses of Hinokiflavone (0–25 μM) for 24 hrs followed by MG132 for an additional 4 hrs (Figure 2d), the levels of both ubiquitinated MDM2 and p53 were noticeably decreased in the presence of Hinokiflavone. Conversely, the levels of unmodified MDM2 and p53 were increased, but only in the cells treated with lower concentrations of Hinokiflavone (1.5 and 3 μM). Higher concentrations of Hinokiflavone (>6 μM) decreased both the ubiquitinated and unmodified MDM2 and p53. These results suggest that Hinokiflavone could attenuate MDM2 and p53 ubiquitination in AML-2 cells. Similar inhibitory effects of Hinokiflavone on MDM2 and p53 ubiquitination were also observed in breast cancer MCF7 cells that have wild-type p53, and on MDM2 ubiquitination in leukemia HL60 cells with p53 deletion, further supporting Hinokiflavone’s role as a potential MDM2 inhibitor (Figure S3).

### 2.3. Hinokiflavone Decreases Cancer Cell Cell Viability

**Figure 3:**
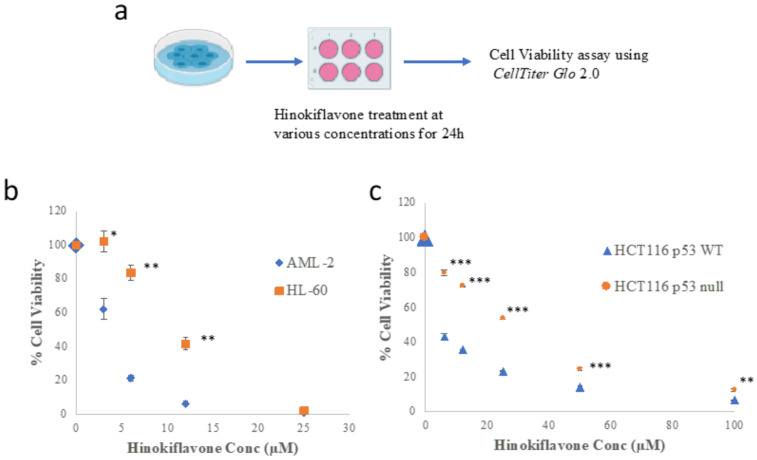
Hinokiflavone decreases cancer cell viability. (**a**) Schematic depiction of Hinokiflavone treatment in the cell cytotoxicity assay. (**b**) AML-2 and HL-60 cells and (**c**) HCT116 and HCT116 p53 null cells were treated with different concentrations of Hinokiflavone for 24 hrs. Cell viability was determined by CellTiter-Glo 2.0. Results were presented as the percentage of cell viability of three different trials. A statistical test was performed with One-way ANOVA. (*) *p*-value < 0.05; (**) *p*-value < 0.01; (***) *p*-value < 0.001.

The cytotoxic effect of Hinokiflavone was evaluated with a panel of cancer cell lines including human leukemia AML-2 and HL-60, osteosarcoma U2OS, breast cancer MCF-7, colorectal cancer HCT116, and a normal human fibroblast cell line BJ (BJ-FB) (Figure 3a, Table 1). Hinokiflavone decreased the viability of all the cancer cells (AML-2, HL-60, U2OS, MCF-7, HCT116) in a dose-dependent manner, with the leukemia AML-2 and HL-60 cells showing the highest sensitivity to Hinokiflavone treatment. In contrast, Hinokiflavone exhibited much lower cytotoxicity in BJ cells, as the BJ cells were viable under Hinokiflavone (0–50 μM) treatment (Table 1, Figure S4).

Next, four cell lines, AML-2, HL-60 cells, HCT116, and HCT116 p53 null, were selected to investigate the p53-dependent and p53-independent cytotoxicity of Hinokiflavone. AML-2 and HCT116 cells encode a wild-type *TP53* gene, whereas HL-60 and HCT116 p53 null cells contain a *TP53* gene deletion. As shown in Figure 3b, although Hinokiflavone demonstrated cytotoxicity in both leukemia cell lines, AML-2 cells were significantly more sensitive to Hinokiflavone treatment compared to HL-60 cells. Likewise, HCT116 cells showed less viability following Hinokiflavone treatment than HCT116 p53 null cells (Figure 3c). The observation that the cancer cells with wild-type p53 were more sensitive to Hinokiflavone suggests that the cytotoxic effect of Hinokiflavone is partially p53-dependent.

### 2.4. Hinokiflavone Decreases the Cellular Levels of MDM2 and MDMX and Induces p53-Dependent and Independent Apoptosis

**Figure 4:**
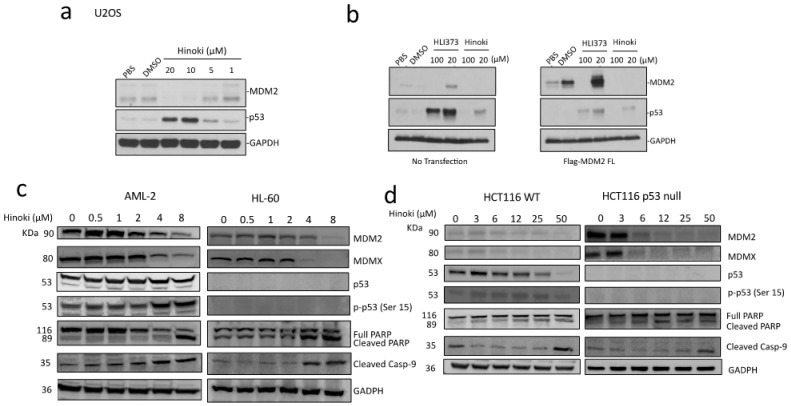
Evaluation of cellular responses to Hinokiflavone treatment in cancer cell lines. (**a**) U2OS cells were treated with various concentrations of Hinokiflavone (0–20 μM) followed by immunoblot detection of MDM2 and p53 protein levels. (**b**) MDM2 and p53 protein levels were detected in U2OS cells treated with various concentrations of Hinokiflavone or HLI373 with or without Flag-tagged MDM2 overexpression. (**c**) AML-2 and HL-60, and (**d**) HCT116 wild-type and p53 null cells were treated with various concentrations of Hinokiflavone for 24 hrs. The levels of MDM2, MDMX, p53, phospho-p53 (Ser15), and apoptosis markers, cleaved PARP and caspase 9, were detected by immunoblotting with the indicated antibodies.

To investigate the ability of Hinokiflavone in activating the p53 pathway, U2OS cells were treated with Hinokiflavone (0–20 μM) and the endogenous p53 and MDM2 levels were determined by immunoblotting. Hinokiflavone induced a dose-dependent p53 accumulation (Figure 4a). Interestingly, Hinokiflavone showed a bimodal effect on MDM2; Hinokiflavone at 1 μM induced a mild increase of MDM2 while moderate to high doses of Hinokiflavone (5–20 μM) resulted in MDM2 downregulation. Compared to the MDM2 E3 ligase inhibitor HLI373 [30], Hinokiflavone treatment at 20 μM resulted in less p53 accumulation and more potent downregulation of MDM2. Hinokiflavone-induced MDM2 reduction was observed with both endogenous MDM2 as well as the transiently transfected Flag-tagged-MDM2 (Figure 4b). Since the ectopically expressed MDM2 was not under the transcriptional control of the endogenous MDM2 promoter, this result suggested that the downregulation of MDM2 by Hinokiflavone could be at the posttranscriptional level.

The cellular responses to Hinokiflavone treatment were further examined using the leukemia AML-2 and HL-60 cells and the colorectal HCT116 cells carrying either wild-type p53 or *TP53* deletion (Figure 4c,d). In both leukemia and colorectal cancer cells, irrespective of p53 status, low doses of Hinokiflavone treatment resulted in a transient and mild increase of MDM2 and MDMX, while moderate to high doses induced a dose-dependent downregulation of both MDM2 and MDMX. In addition, Hinokiflavone treatment induced apoptosis in both leukemia and colorectal cancer cells irrespective of p53 status, exhibiting increased levels of the cleaved PARP and cleaved caspase-9 in a dose-dependent manner (Figure 4c,d).

Since the wild-type p53 cell lines AML-2 and HCT116 were more sensitive to Hinokiflavone treatment, the effect of Hinokiflavone on p53 activation was examined in these cells. Hinokiflavone induced p53 activation was observed by (1) increased p53 phosphorylation at Ser15 in AML-2 and HCT116 cells treated with Hinokiflavone (Figure 4c,d) and (2) by higher mRNA expression of the p53 target genes p21 (*CDKN1A*), *PUMA* and *MDM2* in HCT116 cells treated with Hinokiflavone, but not in HCT116 p53 null cells (Figure S5). These results strongly suggested that Hinokiflavone could induce apoptosis in cancer cells through downregulation of MDM2 and MDMX and activation of p53.

### 2.5. Hinokiflavone Inhibits Cancer Cell Growth

**Figure 5:**
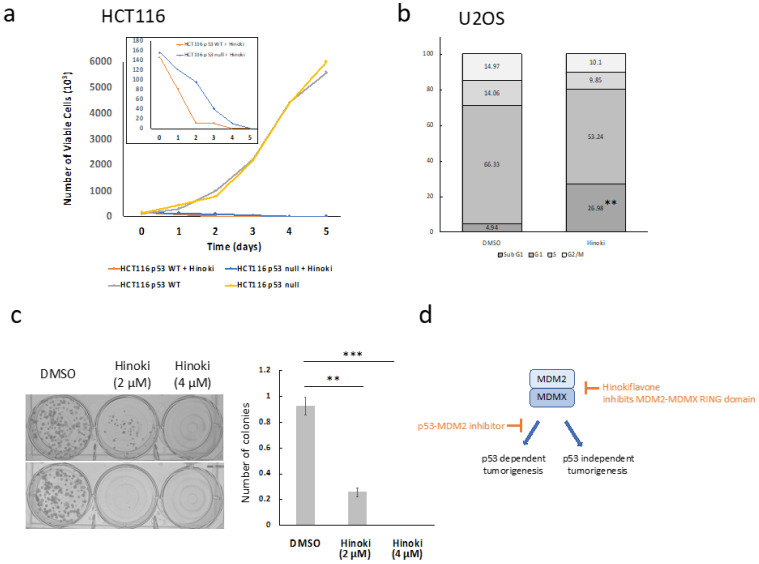
Hinokiflavone inhibits cancer cell proliferation (**a**) Cell proliferation (0–6 days) was plotted for HCT116 cells with wild-type p53 or p53 null treated with 0 µM or 12 µM of Hinokiflavone. The inset image highlights the difference between wild-type p53 and p53 null HCT116 cells following Hinokiflavone treatment. (**b**) Cell cycle analysis by PI staining of U2OS cells treated with Hinokiflavone (20 μM) or DMSO. Results were presented by cell population (%) in sub-G1, G1, S and G2/M using flow cytometry. (**c**) Clonogenic assay of U2OS cells treated with Hinokiflavone. Results were presented as relative colony numbers normalized by the vehicle control (DMSO) (n = 3). Statistical test was performed with two-way ANOVA. (**) *p*-value < 0.01; (***) *p*-value < 0.001. (**d**) Schematic depiction of the anti-cancer potential of Hinokiflavone through inhibition of the MDM2 and MDMX RING domain.

The growth inhibitory effect of Hinokiflavone was further examined in HCT116 cells with either wild-type p53 and p53 null using a cell proliferation assay. As shown in Figure 5a, in the absence of Hinokiflavone, both HCT116 cell lines exhibited exponential growth. Hinokiflavone treatment at a dose close to IC_50_ over the course of six days induced a continuous decline of viable cells. Compared to HCT116 p53 null cells, HCT116 cells with wild-type p53 showed accelerated cell death under Hinokiflavone treatment. Taken together, Hinokiflavone inhibited cancer cell growth irrespective of p53 status, exhibiting greater potency in the wild-type p53 cancer cells.

To evaluate the anti-tumor effects of Hinokiflavone, U2OS cells were treated with 15 μM Hinokiflavone and subject to cell cycle analysis (Figure 5b). Hinokiflavone treatment resulted in a significant increase in the sub G1 cell population, which indicated increased cell death in U2OS cells in response to Hinokiflavone treatment [31]. The inhibitory effect of Hinokiflavone on U2OS cell proliferation was further demonstrated by a clonogenic assay. As shown in Figure 5c, the colony number and size of U2OS cells were significantly reduced when exposed to 2 μM Hinokiflavone, while 4 μM Hinokiflavone completely inhibited U2OS cell colony formation. Collectively, Hinokiflavone exhibited anti-cancer potential in a dose-dependent manner through promoting p53-dependent and independent cell death in MDM2 overexpressing cancer cells.

## 3. Discussion

Small molecule antagonists of p53-MDM2-MDMX interaction have been developed to restore p53 and activate the apoptotic pathway in cancers bearing wild-type p53 [11,32]. Some of the identified p53-MDM2 interaction blockers, represented by AMG232, HDM201, and RG7388, have entered early phase clinical trials [33,34,35,36]. Despite the promise of targeting p53-MDM2 and p53-MDM2 interactions in clinical application, this class of therapeutics showed little effect on the p53-independent oncogenic activity of MDM2 and MDMX. With the increasing understanding of the role of the MDM2-MDMX heterodimer as an active E3 ligase complex in mediating p53-dependent and independent oncogenic activities, targeting the MDM2-MDMX heterodimer presents a unique therapeutic opportunity for anti-cancer drug development. Hinokiflavone was identified using structure-based virtual screening as a candidate compound that showed activity to bind the MDM2-MDMX RING dimer, inhibit the MDM2-mediated ubiquitination, and exhibited p53-dependent and independent anti-cancer effects.

Hinokiflavone induced a bidirectional MDM2 and MDMX response in leukemia and colorectal cancer cells. Low concentrations of Hinokiflavone gave rise to a transient and mild accumulation of MDM2 and MDMX whereas higher concentrations of Hinokiflavone induced downregulation of MDM2 and MDMX. The mechanism underlying the bidirectional effect of Hinokiflavone on cellular MDM2 and MDMX is not clear at present. Since MDM2 was reported to regulate MDMX and its own ubiquitination and degradation [37], inhibition of the MDM2-MDMX RING domain would lead to MDM2-MDMX stabilization [30]. Indeed, MDM2 RING domain inhibitors HLI373 and MEL24 caused MDM2 stabilization [30,38]. However, MDM2-MDMX RING domain inhibitors MMRi64 and analogs induced downregulation of cellular MDM2 and MDMX by disrupting MDM2-MDMX RING domain dimerization [39]. As the Hinokiflavone binding site is juxtaposed with the MDM2-MDMX RING domain dimerization interface in our structural model (Figure 1c and Figure S2), it will be interesting to test whether Hinokiflavone has the activity to destabilize the MDM2-MDMX RING domain complex.

Hinokiflavone induced p53 activation and p53-dependent cytotoxicity in cancer cells. Indeed, p53 stabilization and phosphorylation were observed in a dose-dependent manner in Hinokiflavone treated wild-type p53 leukemia and colorectal cancer cells. Hinokiflavone also exhibited lower IC_50_ values in AML-2 and HCT116 p53 wild-type cells compared to HL-60 and HCT116 p53 null cells, suggesting that the cancer cells that have wild-type p53 were more sensitive to Hinokiflavone treatment. Importantly, Hinokiflavone also exhibited p53-independent anti-cancer activity. Hinokiflavone treatment caused apoptosis and cytotoxicity in cancer cells independent of p53.

Hinokiflavone is a naturally derived bioflavonoid known to possess anti-inflammatory, antioxidant, and antitumor activity [40,41,42,43,44,45]. Several mechanisms have been proposed for the pharmacological activities of Hinokiflavone including inhibition of mRNA spliceosome, activation of mitochondrial ROS/JNK/caspase signaling pathway, as well as inhibition of Matrix Metalloproteinase 9 [40,42,44,46]. Hinokiflavone was previously shown to inhibit tumor growth in colorectal cancer, breast cancer, and melanoma cancer cells in both cell culture and in vivo xenograft models [40,44,45]. In this study, we presented the anti-cancer activity of Hinokiflavone through binding to the MDM2-MDMX RING domain dimer and downregulation of MDM2 and MDMX, which led to p53-dependent and independent cancer inhibition [30,38]. Additional investigation is needed to determine the efficacy of Hinokiflavone in other cancer types and its synergistic anti-cancer potential in combination with other chemotherapy drugs.

## 4. Materials and Methods

### 4.1. In Silico Screening Targeting the MDM2-MDMX RING Domain Dimer

The crystal structure of the MDM2-MDMX RING domain dimer (PDB code: 2VJE) was analyzed for potential “druggable” pockets on the surface of the RING heterodimer complex using PyMOL PocketPicker [47]. Virtual screening was performed using a library of phytochemical compounds by the protein-ligand docking software MGLtools AutoDock Vina to model and evaluate the potential interactions of the small molecules with the binding pocket [47,48,49]. Hinokiflavone was identified as one of the top hitting molecules from this in silico screening (Figure S1).

### 4.2. Cell Lines, Cell Culture, and Antibodies

The cancer cells used in this study were kindly provided by Dr. Sam Benchimol. MCF-7 and BJ cells were grown in Dulbecco’s Modified Eagle’s medium (DMEM) supplemented with 10% FBS. AML-2 cells were grown in MEM Alpha supplemented with 10% FBS. HL-60 cells were grown in RPMI Medium 1640 supplemented with 10% FBS. HCT-116 cells were grown in McCoy’s 5A Medium supplemented with 10% FBS. All cells were incubated at 37 °C with 5% CO_2_.

The primary antibodies used in this study were Ub from Covance (SIG-39400), MDM2 from Santa Cruz, Texas, TX, USA (sc-965), MDMX from Millipore (8C6, 04-1555), p53 from Santa Cruz (DO-1, sc-126), GAPDH from Cell Signaling (#2118), phosphorylated p53 (Serine 15) from Cell Signaling (#9284P), PARP from Cell Signaling (#9542), and cleaved caspase-9 from Cell Signaling (#9505).

### 4.3. Preparation of the MDM2-MDMX RING Domain Dimer

His_6_-tagged MDMX RING domain (residues 416–490) in pET15b and GST-tagged MDM2 RING domain (residues 417–491) in pGEX2TK plasmid were expressed in *E. coli* BL21 DE3 cells at 18 °C using 0.5 mM IPTG. The MDM2-MDMX RING domain heterodimer was prepared by a two-step purification, GST-affinity purification, and subsequently Ni^2+^ affinity purification as described in [50,51]. The purified MDM2-MDMX RING dimer was used in BLI analysis.

### 4.4. Biolayer Interferometry (BLI) Assay

Hinokiflavone interaction with the MDM2-MDMX RING domain dimer was examined using the BLI Octet Red from fortéBIO. The Anti-GST Biosensor (fortéBIO #18-5095, Sartorius, Germany) was loaded with either the GST-tagged MDM2-MDMX RING domain heterodimer or the negative control GST. To quantify the ligand association and dissociation, the biosensor was incubated with different concentrations of Hinokiflavone (0 μM–120 μM) for 400 s in the assay buffer (20 mM MOPS, 50 mM KCl, BSA 0.5 mg/mL). The BLI signal was recorded for a total of 3000 s. The fortéBIO data analysis v9.0 software (fortéBIO, Sartorius, Germany) package was used for data processing and Kd was determined using the one-binding site model.

### 4.5. In Vitro Ubiquitination Assay

Experiments were performed as previously described [51]. The ubiquitination reactions contained 0.1 ug of E1, 0.2 ug of UbE2D2, 5 ug of ubiquitin, and 0.5 ug of E3 (His-tagged MDM2) were performed in a volume of 20 uL for 90 min at 30 °C. Hinokiflavone or MDM2 inhibitor HLI373 (Sigma #373226, Canada) was added to the reaction at the indicated concentrations. Total ubiquitination was detected by immunoblotting using a monoclonal anti-ubiquitin antibody (Santa Cruz, sc-8017). MDM2 ubiquitination was visualized using an MDM2-specific antibody (Santa Cruz, sc-965).

### 4.6. Cell Viability Assay

Cell viability was evaluated using the *Celltiter Glo assays* (Promega Canada, Cat. G9242) following the instructions provided by the manufacturer. Cells were treated with different concentrations of Hinokiflavone for 24 h, mixed with 100 µL of *Celltiter Glo reagent*, and incubated for 10 min at room temperature. Luminescence signal was recorded with the Synergy Hybrid 4 reader. The IC_50_ value of Hinokiflavone was calculated based on the equation $y=Min.+\frac{Max.-Min}{1+{\left(\frac{x}{IC50}\right)}^{Hill\;Coefficient}}$. Statistical analysis was performed based on experimental triplicates through One-Way ANOVA (* *p*-values < 0.05, ** *p*-values < 0.01, *** *p*-values < 0.001).

### 4.7. Detection of Cellular p53 and MDM2 Ubiquitination

Cells were incubated with the indicated concentrations of Hinokiflavone for 24 h. Proteasome inhibitor MG132 10 µM (Millipore Sigma, Mississauga, ON, Canada, Cat. 474787) was added to cells for an additional 4 h post Hinokiflavone-1 treatment. Cells were harvested and lysed using RIPA buffer (50 mM Tris buffer pH 8.0, 150 mM NaCl, 0.5% NP 40) with 1 × protease inhibitor (Roche Cat. 05056489001) and 20 mM N- Ethylmaleimide (NEM, Sigma Canada, Cat. E3876). MDM2 and p53 were detected by immunoblot using the p53 and MDM2 antibodies.

### 4.8. Detection of Cellular Responses to Hinokiflavone Treatment by Immunoblot

U2OS cells were transfected with pc-DNA3.1/Flag-MDM2 plasmid or a control vector using PolyJet transfection reagent (SignaGen Laboratories, Frederick, MD, USA) for 24 h, then treated with Hinokiflavone at various concentrations for 24 h. The levels of MDM2 (Santa Cruz, sc-965) and p53 1 (Santa Cruz, sc-126) were detected by immunoblot using respective antibodies.

AML-2 and HL60 or HCT116 wild-type and p53 null cells were incubated with various concentrations of Hinokiflavone for 24 h. Cells were lysed using RIPA buffer and subject to immunoblotting by specific antibodies for MDM2 (Santa Cruz, Starr County, TX, USA sc-965), MDMX (Millipore Sigma, Mississauga, ON, Canada, 04-1555), p53 DO-1 (Santa Cruz, Starr County, TX, USA sc-126) or phosphorylated p53 at Ser15 (Cell Signaling, New England Biolab Canada #9284P), and cell apoptotic marker protein cleaved PARP1 (Cell Signaling, New England Biolab Canada #9542) and caspase 9 (Cell Signaling, New England Biolab Canada #9505).

### 4.9. DNA Content/Cell Cycle Analysis and Colonogenic Assay

U2OS cells were treated with Hinokiflavone (20 µM) for 16 h and then stained with propidium iodide. DNA content/cell cycle of the treated cells was analyzed by flow cytometry. Cell cycle distribution (%) was calculated based on DNA content, and the results are representative of three independent experiments.

U2OS cells were plated as 6000 cells/per well in a 6-well plate and treated with various concentrations of Hinokiflavone or DMSO for 10 days. Colonies were stained with crystal violet and counted under microscope. The results are presented as relative colony numbers normalized to the cells treated with the vehicle control DMSO, representative of three independent experiments. Statistical analysis was performed through one-way ANOVA (* *p*-values < 0.05, ** *p*-values < 0.01, *** *p*-values < 0.001).

## 5. Conclusions

Hinokiflavone was identified as a plant-based bioflavonoid compound that could interact with the MDM2-MDMX RING domain heterodimer. This study investigated the anti-cancer effects of Hinokiflavone through targeting MDM2. We provided biochemical and cellular evidence suggesting Hinokiflavone is a promising small molecule with anti-cancer potential through inhibition and downregulation of MDM2 in cancer cells.

## Figures and Tables

**Table 1:** Summary of the cytotoxic effect of Hinokiflavone on a selective panel of human cells.

Cell Line	p53 Status	IC_50_ (μM)
AML-2	wildtype	4.93 ± 1.16
HL-60	null	10.95 ± 0.19
HCT116	wildtype	14.19 ± 2.04
HCT116 p53null	null	32.66 ± 0.31
U2OS	wildtype	15.90 ± 2.07
MCF-7	wildtype	17.33 ± 1.90
BJ-FB	wildtype	ND ^1^

^1^ IC_50_ results were represented as mean ± SEM, n = 3. ND, inhibition was not detected under the tested doses.

## Supplementary Materials

The following supporting information can be downloaded at: https://www.mdpi.com/article/10.3390/biom12050643/s1, Figure S1: Structure-based virtual screening strategy and computational prediction of the potential ligand binding sites; Figure S2: The structural model of Hinokiflavone binding to the MDM2-MDMX RING domain dimer; Figure S3: Hinokiflavone attenuated MDM2 and p53 ubiquitination; Figure S4: Hinokiflavone decreases cancer cell viability; Figure S5: Induction of p53 target genes (*CDKN1A, PUMA and MDM2*) in HCT116 wild-type or p53 null cells 24 hours post 6 μM Hinokiflavone treatment; Figure S6: E2~ub charging not affected by Hinokiflavone; Figure S7: The effect of Hinokiflavone treatment on the p21 protein levels in AML-2 and HL-60 cells; Figure S8: The effect of Hinokiflavone on p53 levels in AML-2 cells; Figure S9: The effect of Hinokiflavone on p53 levels in HL-60 cells [52,53,54].
