# Supplementary material for: Hinokiflavone Inhibits MDM2 Activity by Targeting the MDM2-MDMX RING Domain
Source: Biomolecules. 2022 Apr 27;12(5):643. doi: 10.3390/biom12050643 (PMC9138535; doi:10.3390/biom12050643)
Supplement: Supplementary file 1 [file biomolecules-12-00643-s001.zip › biomolecules-1601516-supplementary.pdf]

Hinokiflavone inhibits MDM2 activity by targeting the MDM2-MDMX RING domain

Viktoria K. Ilic [1*] , Olga Egorova [1*], Ernest Tsang [1] , Milena Gatto [1] , Yi Wen [1], Yong Zhao[2] and Yi Sheng[1]

Supplementary Figures

Figure S1. Structure-based virtual screening strategy and computational prediction of the potential ligand binding sites.

Figure S2. The structural model of Hinokiflavone binding to the MDM2-MDMX RING domain dimer.

Figure S3. Hinokiflavone attenuated MDM2 and p53 ubiquitination.

Figure S4. Hinokiflavone decreases cancer cell viability.

Figure S5. Induction of p53 target genes (CDKN1A, PUMA and MDM2) in HCT116 wild-type or p53 null cells 24 hours post 6 μM Hinokiflavone treatment.

Figure S6. E2~ub charging not affected by Hinokiflavone.

Figure S7. The effect of Hinokiflavone treatment on the p21 protein levels in AML-2 and HL-60 cells.

Figure S8. The effect of Hinokiflavone on p53 levels in AML-2 cells.

Figure S9. The effect of Hinokiflavone on p53 levels in HCT116 cells.

Supplementary Methods:

In vitro E2~ub charging analysis

Analysis of the induction of p53 target genes by RT-qPCR

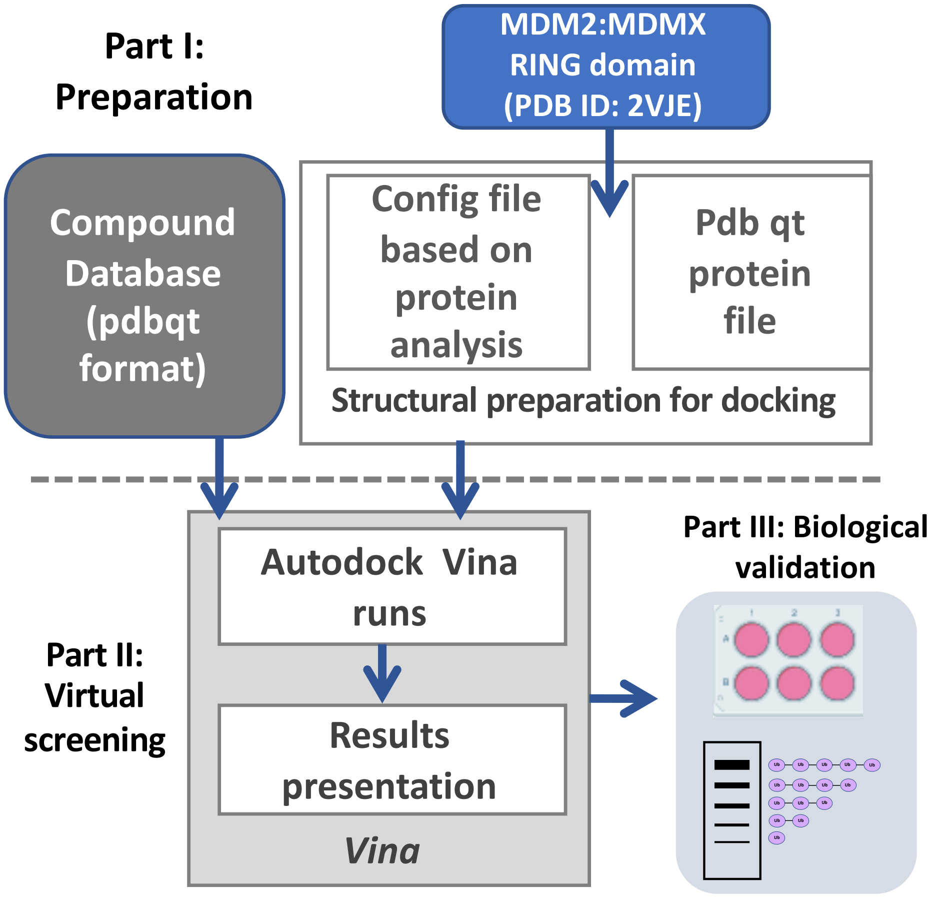

Figure S1 **Structure-based virtual screening strategy and computational prediction of the potential ligand binding sites.** In Part I database of 250 000 natural compounds to be screened is prepared. Crystal structure of the MDM2:MDMX heterodimer (PDB:2VJE) is used to detect potential ligand binding pockets applying PocketPicker software . In Part II virtual screening of the selected ligand library is performed, where each compound within the library is docked into the pocket of interest, and various configurations are tested. In Part III computer generated protein:ligand complexes are analyzed and validated using biological validation.

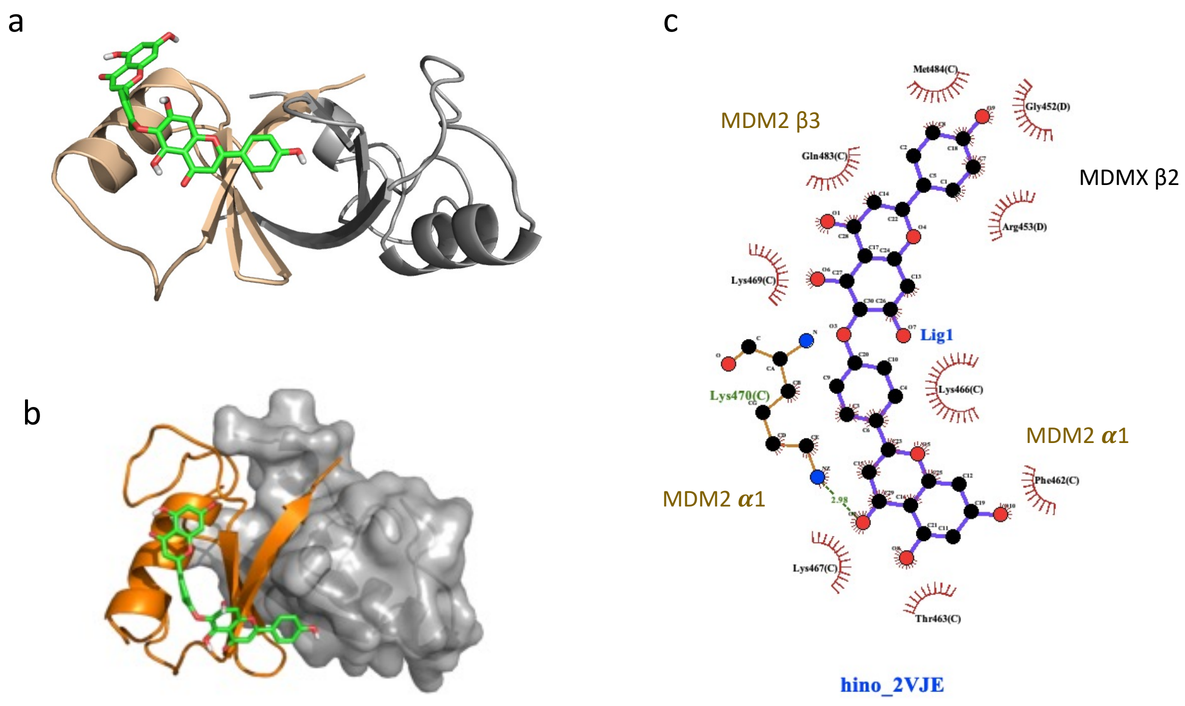

Figure S2. **The structural model of Hinokiflavone binding to the MDM2-MDMX RING domain dimer.** (a) The cartoon representation of the MDM2-MDMX RING domain dimer (2VJE) bound with Hinokiflavone. The MDM2 RING domain and the MDMX RING domain were shown in a ribbon diagram colored in wheat and grey, respectively. Hinokiflavone was shown in the stick form. (b) The MDM2-MDMX RING dimer bound with Hinokiflavone. The MDM2 RING domain was shown as a ribbon representation coloured in or. The MDMX RING domain was shown as a surface rendition colored in grey. Hinokiflavone was shown in the stick form. The MDMX RING domain was depicted in a ribbon diagram and colored in grey. (c) The Ligplot of **the MDM2-MDMX RING domain dimer bound with** Hinokiflavone. The chemical structure of Hinokiflavone was depicted together with the potential interactions with residues from the MDM2-MDMX RING domain dimer. The images were generated with PYMOL[1] and LIGPLOT[2].

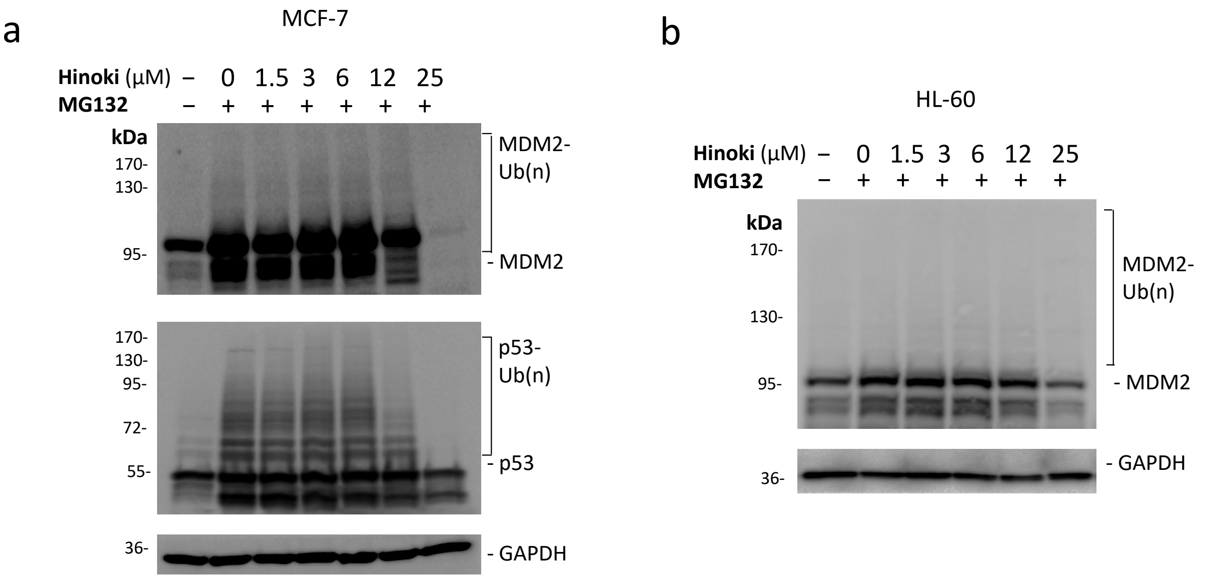

Figure S3. **Hinokiflavone attenuated MDM2 and p53 ubiquitination.** The cells were treated with Hinokiflavone (0 - 25 μM) for 24 h followed by 4 h treatment with protease inhibitor MG132 (10 μM). MDM2 and p53 were detected by immunoblot with MDM2 and p53 specific antibodies. GAPDH was used as a loading control. (a) MDM2 and p53 ubiquitination in MCF-7 cells. (b) MDM2 ubiquitination in p53 null HL-60 cells.

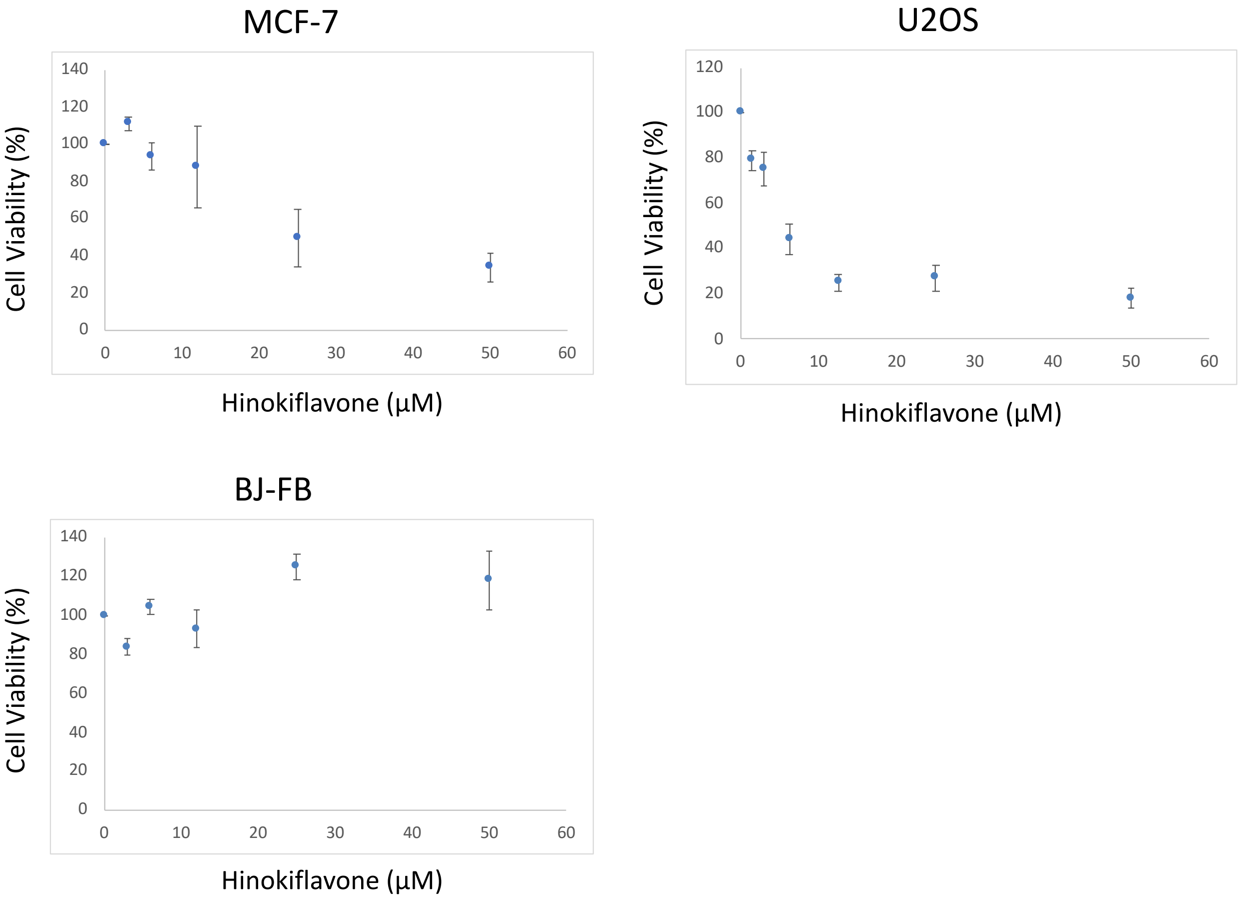

Figure S4. Hinokiflavone decreases cancer cell viability. The effect of Hinokiflavone treatment (0-50 μM) on cell viability of MCF-7, U2OS and BJ cells.

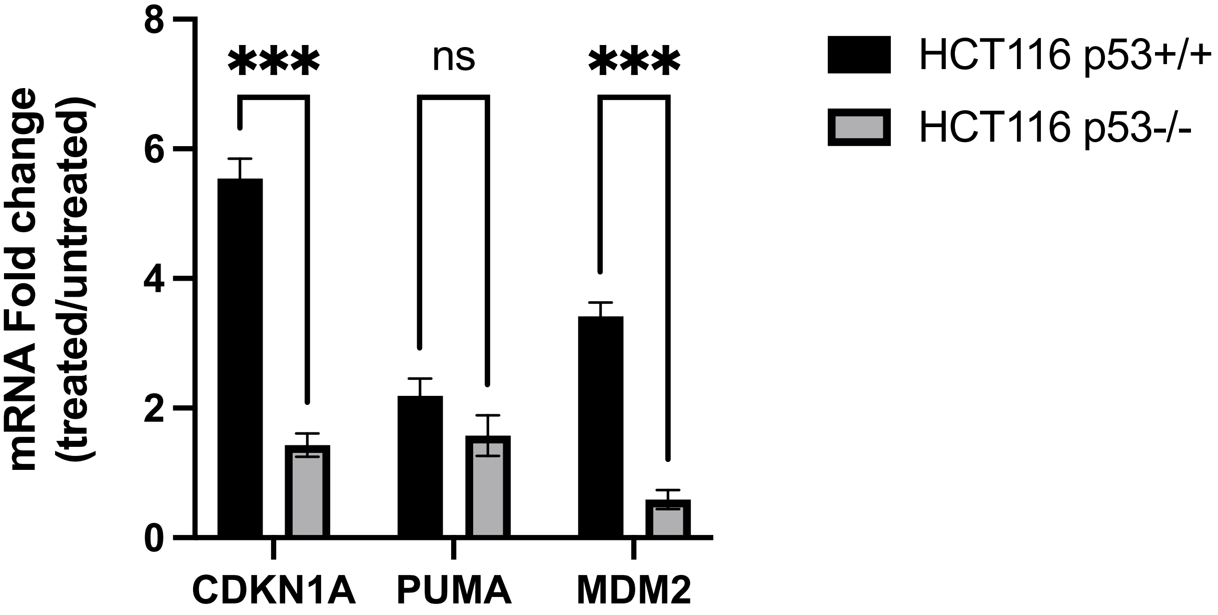

Figure S5. Induction of p53 target genes (CDKN1A, PUMA and MDM2) in HCT116 wild-type or p53 null cells 24 hours post 6 μM Hinokiflavone treatment. mRNA fold change was analyzed by RT-qPCR in HCT116 and HCT116 p53 null cells. Results were presented as relative fold change (rFC) of induction to GAPDH and normalized with untreated cells (n=3). Upregulation of gene expression for CDKN1A, PUMA and MDM2 was shown as rFC. Statistical test was performed with two-way ANOVA. Lowercase letters indicate statistical significance between Hinokiflavone treated and untreated cells (***, p-value < 0.001; ns, not significant).

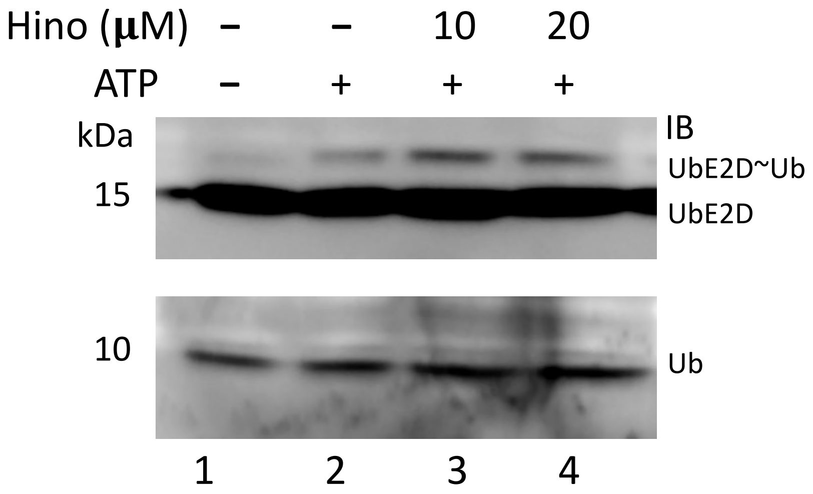

Figure S6. E2~ub charging not affected by Hinokiflavone. The thioester E2~ub intermediate was measured in a reaction containing E1, E2 (UbE2D), and Ub in the presence or absence of Hinokiflavone. ATP was added to initiate the reaction. The proteins were immunoblotted with the antibodies as indicated.

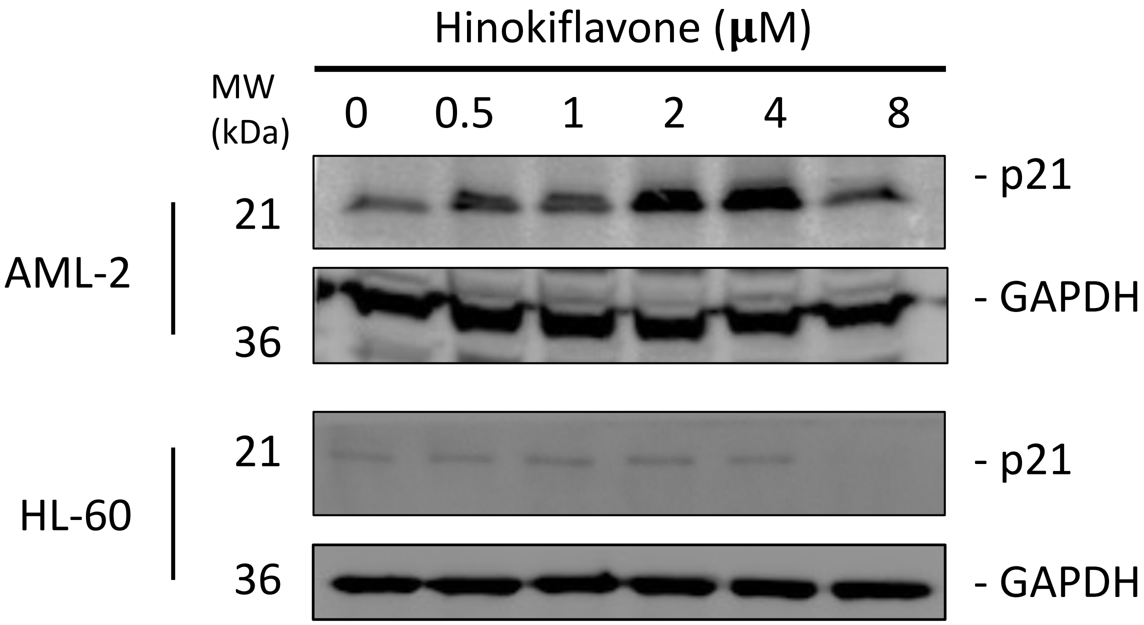

Figure S7. The effect of Hinokiflavone treatment on the p21 protein levels in AML-2 and HL-60 cells. AML-2 and HL-60 cells were treated with various concentrations of Hinokiflavone (0 - 8μM) followed by immunoblot detection of p21. GAPDH was a loading control.

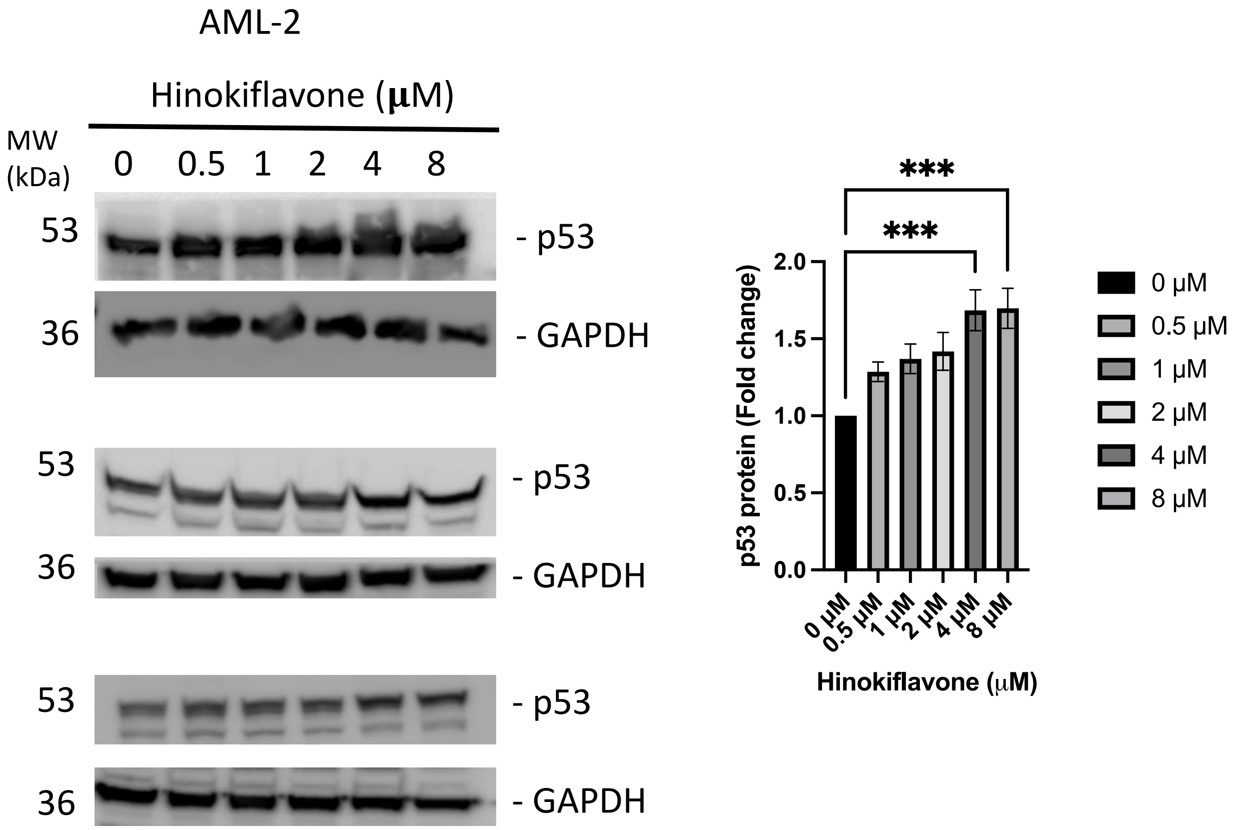

Figure S8. The effect of Hinokiflavone on p53 levels in AML-2 cells. AML-2 cells were treated with various concentrations of Hinokiflavone (0 - 8μM) for 24hrs and subject to immunoblot detection of p53. GAPDH was a loading control. The three independent blots were shown. The effect of Hinokiflavone on the steady state levels of p53 was represented as the fold change normalized using DMSO (vehicle control) treated cells. Statistical analysis was done with Prism using pair-wise T test (***, p value < 0.001).

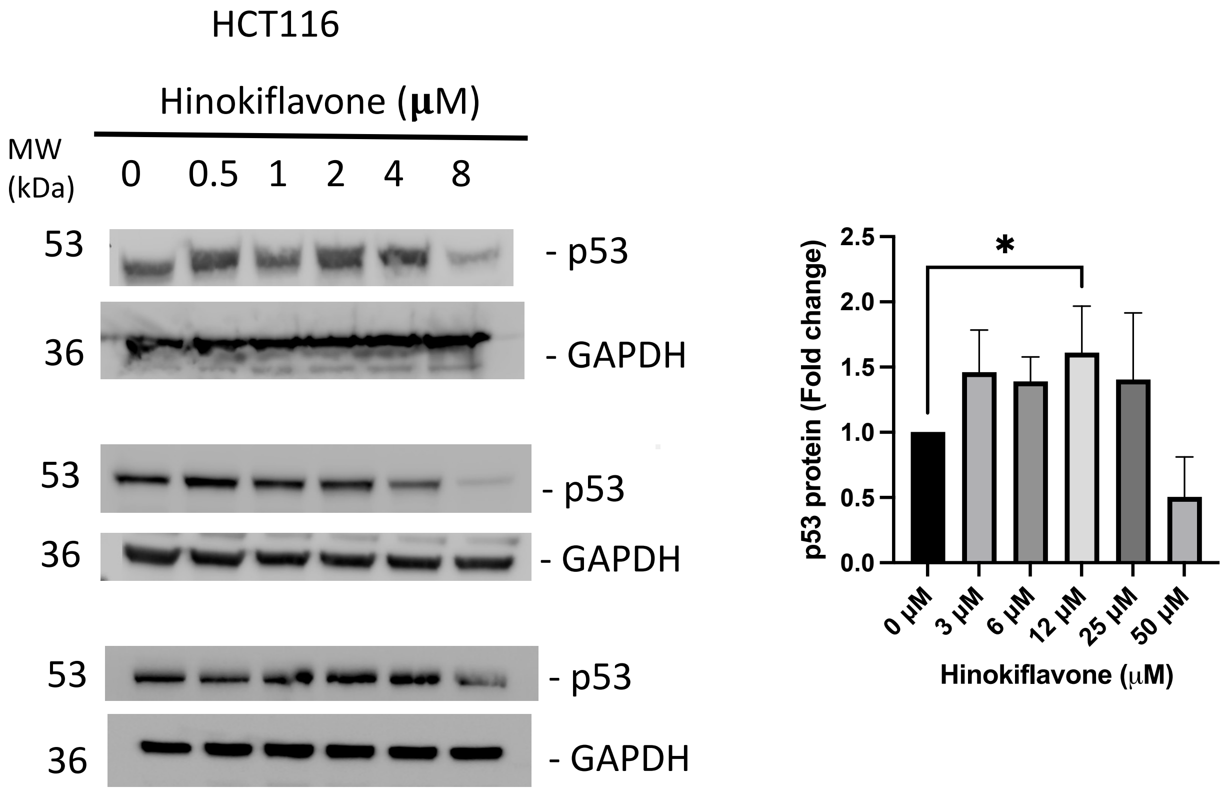

Figure S9. The effect of Hinokiflavone on p53 levels in HCT116 cells. Cells were treated with various concentrations of Hinokiflavone (0 - 50μM) for 24hrs and subject to immunoblot detection of p53. GAPDH was a loading control. The three independent blots were shown. The effect of Hinokiflavone on the steady state levels of p53 was represented as the fold change normalized using DMSO (vehicle control) treated cells. Statistical analysis was done with Prism using pair-wise T test (*, p value < 0.05).

Supplementary Methods:

In vitro E2~ub charging analysis

The thioester E2~ub intermediate was produced in a 20 μL reaction containing E1(0.1μg), E2 (UbE2D, 0.2 μg), and Ub (2 μg) in an assay buffer (10 mM HEPES, pH 7.5, 100 mm NaCl, 40 μM ATP, and 2 mM $MgCl_2$) in the presence or absence of Hinokiflavone (10 or 20 μM). ATP was added to initiate the reaction. The reaction was carried out at 30°C for 10 mins and stopped by addition of SDS sample buffer without DTT. The thioester E2~ub intermediate was detected by immunoblotteing with the E2 specific antibody and Ub antibody[3].

Analysis of the induction of p53 target genes by RT-qPCR

HCT116 wildtype and p53 null cells were treated with 6 μM Hinokiflavone for 24 hours. For RT-qPCR, total RNA was extracted using TRizol (Thermo Fisher). cDNA was synthesized using cDNA reverse transcription kit (Applied Biosystem CAT4373967). Real-time PCR reactions were performed using SsoFast EvaGreen supermix in 20 μl volume. Data were presented as mRNA fold change relative to the control untreated cells from three experimental triplicates. Statistical analysis was performed through the pairwise Student T-test and one-way ANOVA.

Real-time PCR Primer sequences are provided in the table below.

Gene Name	Primer Sequence	Comment
CDKN1A (p21)	5' AGGTGGACCTGGAGACTCTCAG	qPCR_Forward primer
CDKN1A (p21)	5' TCCTCTTGGAGAAGATCAGCCG	qPCR_Reverse primer
PUMA	5' ACGACCTCAACGCACAGTACGA	qPCR_Forward primer
PUMA	5' CCTAATTGGGCTCCATCTCGGG	qPCR_Reverse primer
MDM2	5' TGTTTGGCGTGCCAAGCTTCTC	qPCR_Forward primer
MDM2	5' CACAGATGTACCTGAGTCCGATG	qPCR_Reverse primer
GAPDH	5' AAGGTCATCCCTGAGCTGAAC	qPCR_Forward primer
GAPDH	5' ACGCCTGCTTCACCACCTTCT	qPCR_Reverse primer

Reference for Supplementary material.

1. Lill, M.A.; Danielson, M.L. Computer-aided drug design platform using PyMOL. *Journal of computer-aided molecular design* **2011**, *25*, 13-19, doi:10.1007/s10822-010-9395-8.
2. Laskowski, R.A.; Swindells, M.B. LigPlot+: multiple ligand-protein interaction diagrams for drug discovery. *J Chem Inf Model* **2011**, *51*, 2778-2786, doi:10.1021/ci200227u.
3. Sarkari, F.; Wheaton, K.; La Delfa, A.; Mohamed, M.; Shaikh, F.; Khatun, R.; Arrowsmith, C.H.; Frappier, L.; Saridakis, V.; Sheng, Y. Ubiquitin-specific protease 7 is a regulator of ubiquitin-conjugating enzyme UbE2E1. *J Biol Chem* **2013**, *288*, 16975-16985, doi:10.1074/jbc.M113.469262.
